# CRISPR‐based approaches for studying inborn errors of immunity

**DOI:** 10.1002/ctm2.70021

**Published:** 2024-10-06

**Authors:** Joey H. Li

**Affiliations:** ^1^ Department of Microbiology Immunology, and Molecular Genetics David Geffen School of Medicine at UCLA Los Angeles California USA; ^2^ Molecular Biology Institute University of California Los Angeles Los Angeles California USA; ^3^ Medical Scientist Training Program David Geffen School of Medicine at UCLA Los Angeles California USA

**Keywords:** CRISPR, inborn errors of immunity, neurodevelopmental disorders, NK cells

1

Inborn errors of immunity (IEI), formerly referred to as primary immunodeficiencies, affect millions of children worldwide.^1^ Patients with IEI harbour germline mutations in genes responsible for immune system development or function, resulting in heightened susceptibility to infections as well as non‐infectious sequelae such as increased incidence of malignancy or paradoxical autoimmunity.^1^ While genetic testing is now a standard component of the workup for IEI, interpretation of results remains limited by our knowledge of causal pathogenic variants.^2^ Currently, the primary method of identifying new variants associated with IEI depends on the clinical identification of a patient both bearing a novel mutation and presenting with severe or recurrent infections, pointing to a potential immune defect. While this approach has allowed us to greatly expand our identification of immunodeficient patients, the dependence on a chance encounter with a new patient represents a major barrier to identifying and providing prophylactic care for patients with unrecognized IEIs. Furthermore, this approach is limited to retrospectively identifying immunodeficient patients after they have already suffered from severe or recurrent infections. Therefore, a prospective laboratory‐based screening method to identify putative IEI‐associated genes followed by clinical validation of predicted pathogenic variants could improve our care for immunodeficient individuals.

To test a laboratory‐based approach to prospectively identify and validate new IEI‐associated gene variants, we focused on human natural killer (NK) cells and performed functional knockout screening of developmentally expressed transcription factors using CRISPR.^3^ NK cells play a critical role during the early defense against viral infection via direct cytotoxicity against infected cells as well as the production of inflammatory mediators like interferon (IFN)‐γ. This is highlighted by the increased and often fatal susceptibility to viral infection displayed by NK cell‐deficient individuals.^4^ However, the transcriptional regulators of human NK cell function that could lead to primary NK cell immunodeficiency when mutated remain poorly understood. We applied a CRISPR‐Cas9 ribonucleoprotein (cRNP) electroporation protocol previously optimized by our group for primary immune cells to directly examine the role of 31 distinct transcription factors in mature primary human peripheral blood mononuclear cell (PBMC)‐derived NK cells and identified a single gene, *MEF2C*, required for all tested effector functions.^3^, ^5^ *MEF2C* knockout resulted in defective NK cell proliferation, cytotoxicity against tumor cells, degranulation, and production of inflammatory cytokines. We validated these findings by studying a small clinical cohort of two pediatric patients bearing pathogenic *MEF2C* point mutations resulting in MEF2C haploinsufficiency syndrome (MCHS), a recently characterized neurodevelopmental syndrome with no known associated immune defects.^6^ Neither patient had known immune disorders but reported clinical histories of recurrent infections to varying degrees. Both patients displayed defective peripheral NK cell maturation with decreased mature cytotoxic cells identified as CD56^dim^CD16^+^, and we also observed deficient killing and production of the antiviral cytokine IFN‐γ within the CD56^dim^CD16^+^ subset. Due to the rarity of MCHS patients, we confirmed that *MEF2C* disruption is sufficient to induce NK cell‐intrinsic functional defects by using Cas9 base editing to introduce a patient‐like *MEF2C* point mutation in healthy donor NK cells. This approach phenocopied MCHS patient and *MEF2C* knockout NK cells, with base edited cells displaying defective proliferation and effector functions. Mechanistically, MEF2C was required to activate sterol regulatory element binding protein (SREBP)‐dependent lipid metabolism in response to interleukin‐15/mammalian target of rapamycin (mTOR) complex 1 signaling, and supplementation of CRISPR cRNP‐edited or MCHS patient NK cells with the fatty acid oleate restored cytolytic activity *ex vivo*.

Our findings suggest that the neurodevelopmental disorder MCHS unexpectedly presents with NK cell defects. Case reports suggest MCHS patients may display increased susceptibility to infection, but the cause of these recurrent infections is unknown.^7^ Other scenarios of combined neurodevelopmental and NK cell defects suggest that the central nervous system and immune compartment may share key developmental programs despite originating from distinct embryonic germ layers. For example, patients with heterozygous mutations in *BCL11B*, autism spectrum disorders, or MeCP2 duplication/triplication syndrome have all been reported to present with NK cell developmental arrest, functional impairment, and defects in other immune compartments.^8^, ^9^, ^10^ Mutations in multiple positive regulators of NK cell function identified by our initial CRISPR screen (*KLF3*, *NR4A2*, *SETBP1*, *ZEB2* [Mowat‐Wilson syndrome] and *RORA*) are also associated with reported intellectual disability or neurodevelopmental disorders yet lack formally reported immune defects, suggesting that these gene variants may also represent unrecognized combined neurological and immune deficiency.^3^ Mechanistically, recent studies suggest that type 1 cytokines like IFN‐γ produced by meningeal T and NK cells can modulate brain circuitry to affect behaviour and seizure susceptibility by modulating inhibitory neuronal circuit activity such as homeostatic inhibitory GABA‐ergic signalling in the prefrontal cortex.^11^, ^12^ Immune deficiencies associated with pediatric neurological disorders may be particularly undertreated, as frequent infections in these patients could be mistakenly attributed to increased oral and pharyngeal secretions and impaired clearance rather than innate immune defects.^13^ Therefore, further study will be crucial to better identify and care for these potentially immunodeficient patients.

To our knowledge, non‐viral CRISPR screening of transcription factors has not previously been performed in primary human NK cells prior to this study, though a similar cRNP‐based approach has been applied to primary human T cells to identify new transcriptional drivers of regulatory T cell function.^14^ Additionally, our approach provides a foundation for applying Cas9 base editing to understand cell‐intrinsic roles of single gene defects when patient samples are limited. Identifying sufficient numbers of patients bearing a specific point mutation can be difficult, particularly with rare IEIs. To overcome these clinical limitations, Cas9 base editing can be applied to model the effects of rare patient point mutations at larger sample sizes by using healthy donor PBMCs. While studies using base‐edited PBMCs are limited to examining gene function in mature peripheral immune cells, the effects of gene variants on immune cell development can instead be examined by base‐editing CD34^+^ hematopoietic stem cells. This approach has been previously performed to identify new gene variants that disrupt hematopoiesis as well as therapeutically to correct hematologic disorders like sickle cell anaemia or CD3δ severe combined immunodeficiency.^15^, ^16^ New techniques to overexpress genes directly from the endogenous promoter using CRISPR activation (CRISPRa) offer another angle to interrogate gene function, either through direct overexpression of the affected gene or overexpression of downstream targets or enzymes to rescue the original defect. For example, while we show that oleate supplementation can restore MEF2C‐deficient NK cell function, overexpression of the key oleate‐producing enzyme stearoyl‐CoA desaturase (SCD) could potentially also rescue MCHS‐associated NK cell defects. While the lentiviral introduction of CRISPRa has been used to examine gene function in primary human T cells, non‐viral CRISPRa expression offers an attractive alternative in immune cell types such as NK cells that are more resistant to viral transduction.^17^, ^18^ Together, these CRISPR tools applied to primary human immune cells have high potential to reveal the pathogenicity of putative gene variants associated with inborn errors of immunity while overcoming the challenges of identifying and recruiting large numbers of rare patients (Figure 1).

**FIGURE 1:**
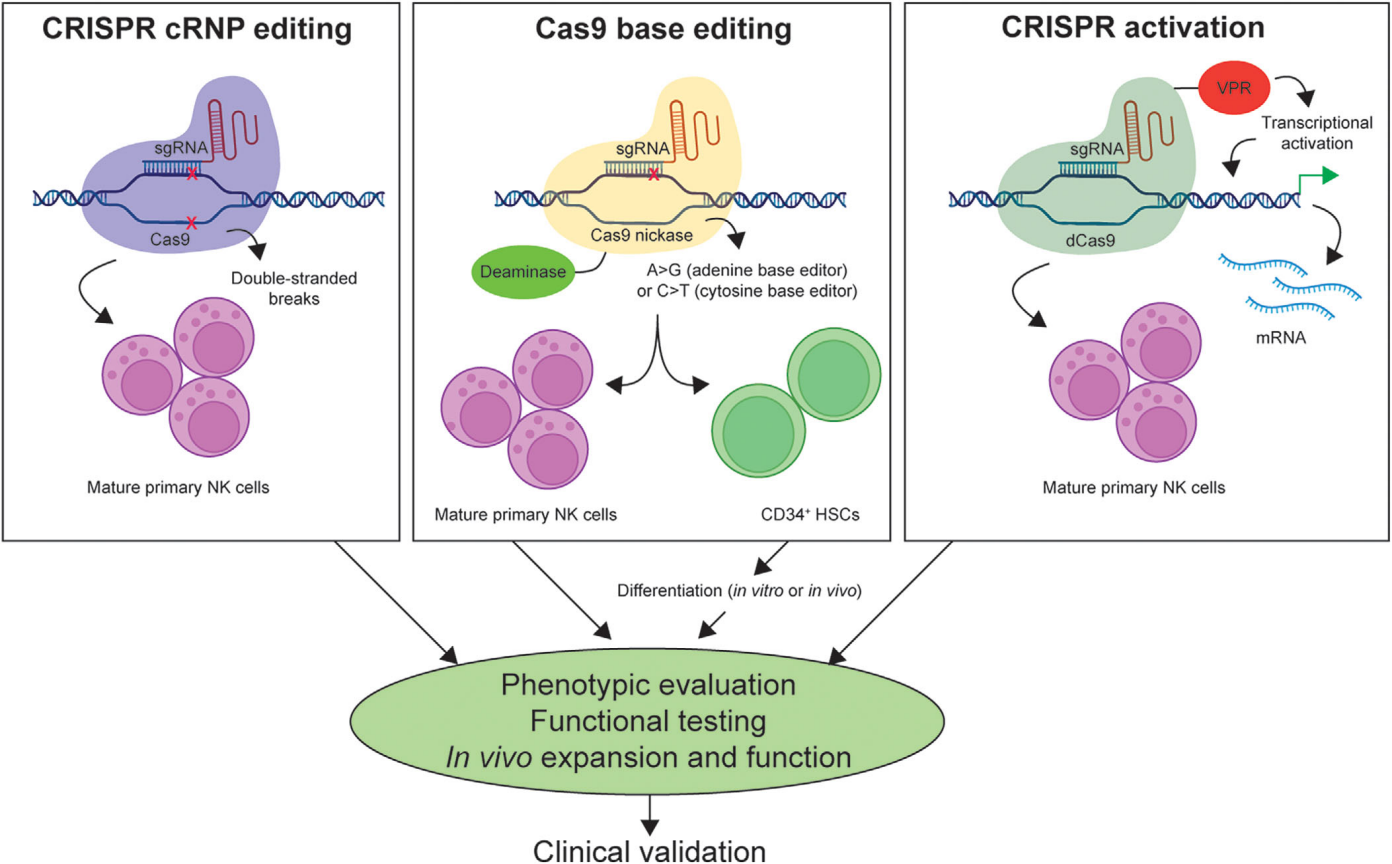
CRISPR‐based approaches to studying primary human immune cell function and development.

As our clinical toolbox for treating immune disorders evolves, identifying more patients with IEI will become increasingly critical to effectively bring these therapies to bear. Our findings highlight the utility of targeted CRISPR cRNP screening and base editing in primary human immune cells to accurately predict and clinically validate a new NK cell immunodeficiency, an approach that can likely be leveraged in other primary human immune cells together with advanced CRISPR tools to identify new IEIs.

## AUTHOR CONTRIBUTIONS

N/A

## CONFLICT OF INTEREST STATEMENT

The author declares no conflict of interest.

## ETHICS STATEMENT

N/A
